# Supplementary material for: Patterns of Intron Gain and Loss in Fungi
Source: PLoS Biol. 2004 Nov 30;2(12):e422. doi: 10.1371/journal.pbio.0020422 (PMC532390; doi:10.1371/journal.pbio.0020422)
Supplement: Table S1 — Also available at http://genes.mit.edu/NielsenEtAl/. (4.3 MB ZIP). [file pbio.0020422.st001.zip › NielsenEtAl/html/541.html]

AN6077.1.NCU01169.1.MG07301.1.FG10231.1


```
 CLUSTAL W (1.82) Multiple Sequence Alignments - Introns Inserted


Sequence 1: NCU01169.1	263 aa
Sequence 2: MG07301.1	253 aa
Sequence 3: FG10231.1	260 aa
Sequence 4: AN6077.1	270 aa
Alignment Length: 278 aa
Number Identitical Residues: 154 aa
Alignment Score (without introns) 6900


MG07301.1 	----MTP-LIRTAVRSACRSTPK-SQISMSSRTISSTASRR-SDTLMV0HRNTPDNNPDI
NCU01169.1	MATKLTPFLMRTAVRAATRLSTKPSTIAPVSRACLSISARRPSDTLMV0HRNTPDNNPDI
FG10231.1 	MASRFTP-LIRSVIRPACRVARP------QSRAAFSLTASRRSDTLMV0HRNTEDNNPDI
AN6077.1  	MASKFFPALPRAGRQFTRQISRP-------QFRPFSAAPQRLSDTLHV0HRNTPENNPSI
          	 :: : * * *:  : : : :         .    * :. * **** * **** :***.*

MG07301.1 	PFKFSAQNEKVITEVLKRYPPQYKKAAVMPLLDIGQRQHGFTSISVMNEVARILEMPPMR
NCU01169.1	PFKFSADNEKVIEEIIKRYPPQYKKAAVMPLLDLGQRQHGFCSISVMNEVARLLEMPPMR
FG10231.1 	PFKFNAENQKVMAEILKRYPPQYKKAAVMPLLDLGQRQHGFTSISVMNEVARLLEMPPMR
AN6077.1  	PFKFSEQNQQLIEEIIARYPPQYKKAAVMPLLDLGQRQHGFTSISVMNEVARILEMPPMR
          	****. :*:::: *:: ****************:******* **********:*******

MG07301.1 	VYEVASFYTMYNRTPVGKYFVQACTT0TPCQLGGCGSDAIVKAIKEELGISQGQTTPDGL
NCU01169.1	VYEVASFYTMYNRTPVGKFHVQACTT0TPCQLGGCGSDVIVKAIKEHLGIKQGETTPDGL
FG10231.1 	VYEVASFYTMYNRTPVGKYFVQICTT0TPCQLGGCGSDVIVKAIKEELGIEQGQTTADGL
AN6077.1  	VYEVATFYTMYNREPVGKYFVQLCTT0TPCQLGGCGSDKIVKAITEHLGITPGHTTEDGL
          	*****:******* ****:.** *** *********** *****.*.***  *.** ***

MG07301.1 	FTFIEVECLGACVNAPMIQINDDYYEDLTPETTKSLLAGLKDPSKAV-------PPAGPL
NCU01169.1	FTFIEVECLGACANAPMVQINDDYFEDLTPETIKQVLSALKESVTDVS----KAPQPGPQ
FG10231.1 	FTILEVECLGACVNAPMIQINDDYYEDLTPASVKDLLKSLRSKATATDPSTVNVPKPGPL
AN6077.1  	FTFIEVECLGACVNAPMVQINDDYYEDLTPESIKELLTALKESATATSG-QVKIPAPGPL
          	**::********.****:******:***** : *.:* .*:.  . ..    . * .** 

MG07301.1 	SGRHSCEHSGGLTNLTSEPWGIETT-RSDL----------
NCU01169.1	SGRQTCENAAGLTSLTSEPYGPEVT-RSDL----------
FG10231.1 	SGRKDCENSAGLTSLTSEPWGTETT-RKDL----------
AN6077.1  	SGRISCENSAGLTNLHNPVWDPETMMRKDGALDGEAQQAQ
          	***  **::.***.* .  :. *.  *.* : ...:..:.
```
